# Serotonin Levels and Cognitive Recovery in Patients with Subacute Stroke after Rehabilitation Treatment

**DOI:** 10.3390/brainsci11050642

**Published:** 2021-05-15

**Authors:** Mariacristina Siotto, Marco Germanotta, Massimo Santoro, Valeria Cipollini, Giulia Guardati, Dionysia Papadopoulou, Elisa Bray, Alessia Mastrorosa, Irene Aprile

**Affiliations:** 1 IRCCS Fondazione Don Carlo Gnocchi ONLUS, 50143 Florence, Italy; msiotto@dongnocchi.it (M.S.); vcipollini@dongnocchi.it (V.C.); gguardati@dongnocchi.it (G.G.); dpapadopoulou@dongnocchi.it (D.P.); ebray@dongnocchi.it (E.B.); amastrorosa@dongnocchi.it (A.M.); iaprile@dongnocchi.it (I.A.); 2 Division of Health Protection Technologies ENEA-Italian National Agency for New Technologies, Energy and Sustainable Economic Development, 00123 Rome, Italy; massimo.santoro@enea.it

**Keywords:** post-stroke depression, post-stroke cognitive impairment, serotonin, 5-HT, stroke, rehabilitation, cognitive functions, SSRIs

## Abstract

Post-stroke depression and cognitive impairment are common conditions affecting patients after stroke. Serotonin is a neurotransmitter involved in modulating, among others, mood, cognition, learning, and memory. Sub-optimal serotonin activity may be in part responsible for cognitive deficits seen in depression. In this pilot study serotonin levels were evaluated in 29 patients with sub-acute stroke before and after a rehabilitation treatment (consisting of a program of upper limb robotic rehabilitation in addition to conventional physical therapy treatment). We employed the Back Depression Inventory scale to evaluate symptoms of depression, and specific tools to evaluate cognitive functions. We found a significant reduction of the serotonin levels after rehabilitation in the whole group (T0: 85.9 ± 92.4 ng/mL; T1: 61.9 ± 58.4 ng/mL; *p* = 0.0018), as well as in the subgroup of patients untreated with Selective Serotonin Reuptake Inhibitors (SRRI), (mean serotonin at T0: 154.0 ± 102.3 ng/mL; mean serotonin at T1: 92.9. ± 68.7 ng/mL at T1; *p* = 0.005). We also found a correlation with cognitive assessment: in particular, the change from baseline of the serotonin (ΔSerotonin) was correlated with the changes from baseline of the Rey’s Figure (ΔROCF) (*r* = 0.535; *p* < 0.05), the Tower of London (ΔToL) (subscore point: *r* = 0.621; *p* < 0.005; subscore time: r = −0.619; *p* < 0.005) meaning that a serotonin levels decrease is associated with a worsening of cognitive functions. Considering patients treated and untreated with SSRIs separately, in patients treated with SSRIs (*n* = 16) we found only a positive correlation between ∆Serotonin and ∆ToL (subscore point: *r*= 0.587; *p* = 0.045), whereas in patients untreated with SSRIs (*n* = 13) we found a positive correlations between ΔSerotonin and ΔROCF (*r* = 0.700; *p* = 0.036), ∆Stroop (subscore time: *r* = 0.750; *p* = 0.020) and ∆Tol (subscore point: *r* = 0.740; *p* = 0.023) and a negative correlation between ΔSerotonin and ∆Tol (subscore time: *r*= −0.833; *p* = 0.005). These results suggest that variation of serotonin levels should be monitored in patients during a rehabilitation program, not only for their relationship with depression symptoms, but also for the correlation with cognitive performance.

## 1. Introduction

Stroke is the first cause of disability [1] and the second largest cause of death worldwide, and it has a high burden on patients, their families, and healthcare systems [2].

Post-stroke depression (PSD) and cognitive impairment (PCI) are very common conditions that can involve patients after stroke [3,4,5].

PSD occurs in a noteworthy number of patients and represents an important complication of stroke, leading to greater disability and increased mortality. The frequency of PSD has been extensively studied and the most recent meta-analysis of 61 cohorts including 25,488 patients reported that 31% of patients developed depression at any time point up to 5 years following stroke [6] even if this meta-analysis did not examine the time since stroke and the clinical setting (e.g., community or hospitalized patients) [7]. The pathophysiology of PSD is likely multifactorial, encompassing a combination of various ischemia-induced neurobiological dysfunctions in the context of psychosocial distress [8,9].

PCI is another frequent condition that affects more than 50% of the subject after stroke [10,11,12,13]; it is related to compromised functioning and a higher risk of mortality [14,15].

The relationship between PSD and cognitive impairment has been well established; early antidepressant treatment of PSD appears to enhance both physical and cognitive recovery from stroke and might increase survival up to 10 years following stroke [7]. Cognitive impairment has a significant impact on functional recovery, quality of life, and sociality. Moreover, cognitive impairment could negatively influence rehabilitation strategy [16] and also be a negative predictor of functional and motor outcomes in post-stroke subjects who underwent to upper limb robotic rehabilitation [17]. A recent explorative study from our research group on 51 patients revealed a beneficial effect of robotic upper limb rehabilitation on cognitive functions [18]. In particular, after the robotic rehabilitation program, patients improved in all the investigated cognitive domains, as measured by selected cognitive assessment scales [18]. Moreover, the cognitive impairment seems to influence the improvement of the upper limb motor performance and ability of activities of daily living after rehabilitation [19].

Serotonin (5-hydroxytryptamine, 5-HT) is a biogenic monoamine intermediate product of tryptophan metabolism. Serotonin is a neurotransmitter located primarily in the enterochromaffin cells of the intestine—which account for 80% of the body’s content- but also in serotonergic neurons of the brain, and in blood platelets. Its biological function is multifaceted and complex, modulating mood, cognition, learning, memory, and numerous physiological processes. Low levels of serotonin in the brain have been studied in relation to depression, anxiety, and sleep trouble. Sub-optimal 5-HT activity may be in part responsible for cognitive deficits seen in depression [20]. Moreover, low serotonin function can affect cognitive flexibility, which is the capability to behavioral adaptions to changing environmental demands, and long-term memory functions, attentional functions, such as focused and sustained attention [21]. An exploratory study found that the serotonin concentration both in plasma and in CSF in patients with PSD was considerably lower than in non-depressed patients, measured 30 days following the stroke [22]. Moreover, a more recent research provided some observation that low extracellular 5-HT levels are associated with impaired memory consolidation but suggested that larger, consistently designed studies are necessary to understand the roles of 5-HT in cognition in healthy and depressed individuals [23].

In the light of the above, this study aims to investigate the relationship between serum serotonin levels and cognitive function on a group of patients with subacute stroke undergoing rehabilitation treatment. 

## 2. Materials and Methods

### 2.1. Study Design and Participants

Patients with a first stroke admitted to our rehabilitation department, coming from a hospital or post-acute rehabilitation facilities, between 2019 and 2020, were consecutively enrolled. 

The inclusion criteria were as follows: (i) first ischemic or hemorrhagic stroke, documented by magnetic resonance imaging (MRI) or computed tomography (CT); (ii) age between 55 and 85 years; (iii) patients able to perform a rehabilitation treatment, for at least 45 min/day, for 5 days/week; (iv) time since stroke onset within 6 months; (v) cognitive and language abilities sufficient to understand the experiments and follow instructions. 

The exclusion criteria were as follows: (i) a previous stroke; (ii) behavioral and cognitive disorders and/or reduced compliance interfering with active therapy. 

The study design was approved by the Ethical Committee of Don Carlo Gnocchi Foundation, Milan, Italy on 13 March 2019 (FDG_6_13/3/19). Written informed consent was obtained from all patients after a detailed explanation of the study’s aims and rehabilitation protocols (clinical trials identifier: NCT04223180).

### 2.2. General Assessment

Demographic, anamnestic, and clinical data were recorded at the enrollment. Cognitive functions, upper limb performance, and dependence in daily living activities, as well as serotonin serum level, were assessed before (T0) and after the robotic rehabilitation intervention (T1). Subjects were analyzed for depression with the Beck Depression Inventory (BDI) scale, which is a 21-question multiple-choice self-report inventory psychometric test for measuring the severity of depression [24]. When the test is scored, a value of 0 to 3 is assigned for each answer and then the total score is compared to a key to determine the depression’s severity. The standard cut-off scores were as follows: 0–9: indicates minimal depression; 10–18 indicates mild depression; 19–29 indicates moderate depression; 30–63 indicates severe depression. Patients admitted to the rehabilitation unit with a diagnose of depression were treated with serotonin inhibitors (SSRIs).

### 2.3. Cognitive Assessment

The cognitive assessment included the following tests:

*Symbol Digit Modalities Test* (Attention and processing speed). It is an easily administered test for overall neurocognitive and executive functioning including attention, planning, and organizing in addition to visual scanning, and motor speed. The subject is presented with a page where, in the first row, nine symbols are one-to-one associated with nine digits, from 1 to 9. Then, the rows below contain only symbols, and subjects are required to orally report the digit associates with each symbol. The number of correct responses in 90 s is measured. A higher score indicates higher cognitive functions [25,26].

*Digit Span task* (Memory). We used the Digit span forward task originally proposed by Hebb [27]. The examiner pronounces a list of digits, at a rate of approximately one digit per second, and the subjects are required to immediately repeat the list in the same order. If they succeed, a list one digit longer is presented. If they fail, a second list of the same length is presented. If subjects are successful on the second list, a list one digit longer is given, as before. However, if subjects also fail on the second list, the test is ended. The length of the digit sequences gradually increases, starting with a sequence of three numbers (e.g., 5, 8, 2) to a sequence of a maximum of nine items (e.g., 7, 1, 3, 9, 4, 2, 5, 6, 8). The span is established as the length of the longest list correctly recalled [28]. 

*Rey-Osterrieth Complex Figure* (Visuospatial abilities and Visual memory). The task, originally designed by Rey (Rey 1941) and later standardized by Osterrieth [29], requires the subject to copy a complex geometrical figure (immediate copy condition) [30,31]. For the test, performance accuracy was calculated by applying the standard scoring criteria, in which the geometrical figure is divided into 18 units and scored on a 2-point scale for both accuracy and placement [32].

*Tower of London* (Executive functions). It is a useful neuropsychological instrument to measure planning and problem-solving abilities [33,34,35]. Briefly, it consists of a board with three vertical pegs of different increasing lengths in which three different wooden balls of different colors are placed. The shortest peg only accommodates one ball, the second two, and the third three. Subjects are presented with a given configuration of balls inside the pegs and a picture of the final configuration. Subjects are then required to move the balls to reach the final configuration, without breaking some rules (each peg can accommodate a different number of balls, just one ball might be moved at a time, the balls cannot be placed outside the pegs, and a maximum number of moves is allowed). In this study, three scores were computed: points, time (measured as the sum of the planning and the execution time), and errors [35].

*Stroop Color and Word Test*. (Executive functions). It is a neuropsychological tool widely used in clinical practice to assess selective attention, cognitive flexibility, and sensitivity to interference, abilities which have been linked to the frontal lobes. We used the short version [36] in which three tasks are proposed: (1) word—(word reading): 3 lists of 10 words (“red”, “blue”, “green”) are provided in random order to the patients, each written with black ink; they must read the written words; (2) color—(color designation): 3 lists of 10 colored circles (red, blue, green) are provided in random order to the patients; they must name the color of the circles; (3) color-word (interference test): 3 lists of 10 words (“red”, “blue”, “green”), each written with colored ink (red, blue, or green) different from the name of the color indicated by the word, in all possible combinations, are proposed to the patients in order random, and they are asked to name the color of the ink used to write the word, not the word itself. For each test, the execution time and any errors made are recorded. Two interference effects are then calculated and used as outcomes: time (difference between the time spent in the third test and the average time spent in the two previous tasks), and error (difference between the number of errors made in the third test and the average time spent in the two previous tasks).

The cognitive outcome measures were administered before and after the rehabilitation program.

### 2.4. Rehabilitation Treatment

Patients underwent a rehabilitation program including conventional physical therapy treatment, performed 6 days a week, lasting 45 min, focused on lower limbs (sensorimotor stimulation, passive, active-assisted and active mobilizations, exercises for muscle strength recovery, stretching, and functional and task-oriented training), proprioceptive exercises, postural passages, and transfers, sitting and standing training, motor coordination and balance training, walking training, and activities of daily living recovery training. Moreover, all patients performed a robotic treatment of the upper limb 5 times a week, each session lasting 45 min, using a set of robotic devices. Robotic treatment of the upper limb was based on the use of 4 robotic devices (Motore—Humanware Srl, Pisa, Italy, and Amadeo, Diego and Pablo—Tyromotion GmBH, Graz, Austria). During the upper limb robotic treatment, patients performed both motor and cognitive tasks, and the devices provided visual and auditory feedback to help them. In particular, a set of motor/cognitive exercises was selected among those available in the robotic devices to train attention, memory, executive function, speed of processing, and visuospatial abilities [9].

Patients were evaluated at baseline (T0) and re-evaluated after 6 weeks of rehabilitation treatment (T1) by means of the Fugl-Meyer Assessment for Upper Extremity (FMA-UE) [37], to evaluate motor function; the upper-extremity subscale of the Motricity Index [38], to evaluate upper limb muscle strength; and the Modified Barthel Index [39], to evaluate activities of daily living and mobility.

### 2.5. Serotonin Analyses

The blood samples of patients at T0 and T1 were collected in the early morning (7:30–9:00 a.m.) after an overnight fast in order also to standardize the assessment of those biochemical variables that are affected by the circadian cycle and food intake. Sera samples were separated by centrifugation (3000 rpm, 10 min, and 4 °C). They were then divided into 0.5 mL aliquots and rapidly stored at −80 °C. Subjects’ samples were thawed just before the assay. All the analyses of serum were performed in duplicate. 

Quantitative determination of serum serotonin levels was done by ELISA enzyme Immunoassay fast track test (Mybiosource, San Diego, CA, USA) on an Absorbance microplate reader (iMARK™, BIO-RAD Laboratory Inc., Hercules, CA, USA). In the first step, the serotonin is quantitatively acylated and a subsequent competitive reaction allows the measurement at 450 nm. The normal reference values are 70–270 ng/mL.

### 2.6. Statistical Analysis

Serum serotonin data were normally distributed and then, a parametric analysis was performed. To evaluate the changes of the serum serotonin levels during the rehabilitation intervention on the whole sample, data measured at T0 were compared with those at T1 by means of a paired *t*-test. Moreover, to ascertain whether the pharmacological intervention could impact the variation of the serum serotonin levels, we performed a 2 × 2 mixed Anova test, with time (2 levels: T0 vs. T1) as within factor, and pharmacological intervention (2 levels: treated vs. untreated patients) as between-subject factor, followed by a simple effect analysis, if necessary.

For further analysis, due to the ordinal nature of the clinical scales, non-parametric tests were used. To assess the relationship between the serum serotonin levels and the clinical picture of the patients (in terms of motor ability, QoL, and cognitive impairment) at baseline, we computed the Spearman’s Rank correlation coefficients (for the whole sample, and for treated and untreated patients, separately). Finally, we first computed the change from baseline (i.e., the difference between the post-treatment and the pre-treatment scores, ∆) of the serum serotonin levels and the clinical and cognitive scales; then, we used the Spearman’s Rank correlation coefficients to analyze the relationship between the baseline value and the changes of the serum serotonin levels with the clinical and cognitive improvement.

For all the statistical analyses, a *p*-value lower than 0.05 was deemed significant. Statistical analysis was performed using SPSS (IBM SPSS Statistics for Windows, Version 25.0. Armonk, NY, USA).

## 3. Results

### 3.1. Participants and Baseline Characteristics

For the study, 29 patients were enrolled and evaluated at baseline (T0) and after treatment (T1).

Table 1 reports baseline characteristics (demographic and clinical features) of sample and baseline serum serotonin values. According to the BDI score, patients’ depression was classified as minimal to moderate (BDI lower than 30), or severe (BDI equal to or higher than 30) [25]. Antidepressant Selective Serotonin Reuptake inhibitors (SSRIs) treatment was administered to a total of 16 patients, based on BDI score and/or confirming therapies administered in 11 patients from previous hospital facilities.

### 3.2. Serotonin Changes after Rehabilitation Programs

**Figure 1:**
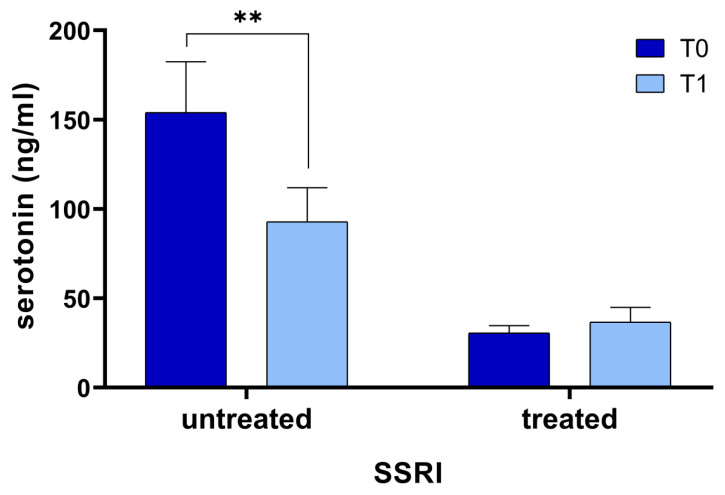
Serotonin serum levels in patients before (T0) and after (T1) the rehabilitation treatment, considering patients untreated and treated with Selective Serotonin reuptake Inhibitors (SSRI), separately. The asterisk indicates a statistically significant difference: ** *p* < 0.005, according to the *t*-test.

The *t*-test revealed a statistically significant difference between serotonin levels at T0 (85.9 ± 92.4 ng/mL) and T1 (61.9 ± 58.4 ng/mL; *p* = 0.018). According to the Anova test, the interaction factor was statistically significant (*p* = 0.001), meaning that the behavior of the two groups over time was different and, therefore, a simple main effect analysis was carried out. In 13 patients untreated with SSRIs we found that serotonin levels at T0 significantly decreased at T1 (T0 = 154.0 ± 102.3 ng/mL; T1 = 92.9 ± 68.7 ng/mL; *p* = 0.005; Figure 1); moreover, 16 patients treated with SSRIs showed very low serotonin serum levels at T0 (30.6 ± 16.5 ng/mL) that did not significantly change at T1 (36.7 ± 32.8 ng/mL; *p* = 0.465).

### 3.3. Correlation Between Serotonin Levels and Rehabilitation and Cognitive Performance Assessment

In the whole group, at baseline, serotonin levels at T0 correlated only with a cognitive test, specifically the subscore point of the Tower of London test (r = 0.583; *p* = 0.006) but not with the motor/disability assessment, as showed in Table 2.

On the contrary, we found that the change from baseline of the serum serotonin (∆Serotonin) correlated with the change from baseline of the Rey’s Figure (∆ROCF), and the change from baseline of two over three Tower of London scores, specifically the point (∆Tol_point), and the Time (∆Tol_time) subscores. In particular, we found a positive correlation between ∆Serotonin and change in scores of ROCF (*r* = 0.535; *p* = 0.013), between ∆Serotonin and change in scores of Tol_point (*r* = 0.621; *p* = 0.003) and a negative correlation between ∆Serotonin and change in time scores of Tol_time (*r* = −0.619; *p* = 0.003) (Table 3).

Considering patients treated and untreated with SSRIs separately, we analyzed correlations of ∆Serotonin with changes in cognitive and in motor assessment reported in in Table 4. In particular, in treated patients (*n* = 16) we found only a positive correlation between ∆Serotonin and ∆Tol_point (*r* = 0.587; *p* = 0.045); in untreated patients (*n* = 13) we found a positive correlations between ∆Serotonin and ∆ROCF (*r* = 0.700; *p* = 0.036), ∆Stroop_time (*r* = 0.750; *p* = 0.020) and ∆Tol_point (*r* = 0.740; *p* = 0.023) and a negative correlation between ∆Serotonin and ∆Tol_time (*r* = −0.833; *p* = 0.005) (Table 4).

## 4. Discussion

The principal aim of this study was to investigate the possible correlation between systemic serotonin levels and cognitive impairment in patients with subacute stroke undergoing rehabilitation treatment.

First of all, we obtained an important finding: patients untreated with SSRIs during the six-week rehabilitation program had a decrease in their serum serotonin levels (T0 = 154.0 ± 102.3 ng/mL; T1 = 92.9 ± 68.7 ng/mL; *p* = 0.005; Figure 1).

It is important to note that we measured only serum levels of serotonin, which may not reflect the serotonin really acting in the brain; however, Gao et al., measuring 5-HT in plasma and in CSF 30 days following the stroke, found serotonin concentration considerably lower in patients with PSD than in non-depressed ones [22].

A potential explanation for the decrease in serum serotonin levels could be that the tryptophan, which is the precursor of serotonin, could be degraded preferring the kynurenine pathway. This pathway has been studied in several depression disorders such as Major depressive Disease, as reported in the literature [40]. In our study, we did not measure serotonin or tryptophan metabolites and this prevents us from making assumptions.

Although a direct role of serotonin in depression is still a matter of debate, we can suppose that this result is probably due to a progressive increase in depressive symptoms during hospitalization. In fact, generally, patients’ mood worsens in the months following the onset of the stroke because patients develop a progressive awareness about his/her disability. Moreover, the hospitalization for several weeks is itself a cause of depression. Unfortunately, we did not record BDI after rehabilitation treatment and we did not have a control group to compare the data; therefore, we can only hypothesize this explanation. Depression characterizes actually about 30% of patients in the acute and medium-term phases, with an increase in the long-term phase of recovery after stroke [41]. Note that investigating the differences between patients treated with SSRI and untreated patients, we found that in patients treated with SSRIs the serum serotonin levels at T1 did not change, remaining always below normal levels, while in untreated patients serotonin dropped significantly (Figure 1). Therefore, we believe that untreated patients should be monitored with major attention concerning possible development of depression during the hospitalization for rehabilitation. It is important to underline that the assessment of depression in stroke survivors may be often laborious and the risk of inappropriate diagnosis (under- or over-diagnosing) is high [42]. In fact, PSD may not only be over-diagnosed because of somatic symptoms caused by medical illness but also under-diagnosed, particularly in patients with cognitive impairment. Moreover, patients with mild depression sometimes refuse pharmacological treatment with antidepressive drugs.

The analysis of cognitive assessment with cognitive tests at admission (T0) and after the rehabilitation program (T1), allowed us to analyze the correlation with the change of serum serotonin levels at T0 and at T1 (ΔSerotonin). This preliminary study showed that a positive correlation exists between ΔSerotonin and ΔROCF. We found that the decrease of serotonin serum levels is associated with a change in scores of the ROCF, which is a useful cognitive test for monitoring visuospatial abilities and visual memory.

The ToL is a test widely employed for cognitive evaluation that measures planning and problem-solving abilities; a positive correlation was found between ∆Serotonin and change in scores of Tol_point and a negative correlation between ∆Serotonin and change in time scores of Tol_time. This means that patients with a decrement in serotonin, tended to have worse performance, with a reduction in the point score and a corresponding increase in the time necessary to perform the test.

Moreover, dividing patients in treated and untreated with SSRIs, we obtained that untreated patients showed very strong correlations between changes in serotonin and in cognitive functions, being that the decrease in serotonin is correlated with a worse performance in the ROCF and the Tol (points and time subscores); treated patients showed only a correlation between decrement in serotonin and decrement in Tol_points.

The number of patients analyzed was low but from our preliminary study appeared that the variation of serum serotonin levels should be carefully monitored in subacute stroke patients in order to avoid a worsening of cognitive performance during hospitalization for rehabilitation; this should be done especially in patients apparently less depressed or not treated with SSRIs in which apparently there is a correlation between serotonin serum level and the cognitive impairment.

The correlation between serotonin and cognitive function is in line with the biochemical function of serotonin. In fact, regarding brain function has been shown that low brain serotonin levels are associated with poor memory and depressed mood [43]. Reduced serotonin turnover is consistently associated with impaired long-term memory functioning being that low serotonin function may also impair cognitive flexibility [20]. Moreover, the association between mood, food, and cognition has been recently underlined [44]. The general biochemical and nutritional status of patients under rehabilitation is becoming an important issue to carefully monitor [45,46,47]. In our rehabilitation center, we found that total serum calcium was lower than normal in about 30% of patients, together with low levels of total protein in about 70% of patients [48]. Moreover, recently we measured very low serum 25-OH vitamin D levels in almost all our patients (data not published); these results suggest a compromised nutritional status of patients hospitalized after stroke. Brain serotonin is synthesized from tryptophan by tryptophan hydroxylase 2, which is transcriptionally activated by vitamin D hormone. Inadequate levels of vitamin D (~70% of the population) and omega-3 fatty acids are very common, suggesting that brain serotonin synthesis is not optimal [49]. Although purified tryptophane increases serotonin, foods containing tryptophane do not [50], so it is not easy rise serotonin levels only with diet. Increasing positive emotions, exposition to bright light, and exercise have been addressed as possible non-pharmacologic methods of raising brain serotonin [50], but in case of important dropping, it is necessary to resort to pharmacological treatment with antidepressive drug as SSRIs.

The limitation of this study is the relatively small sample size, which advocates the necessity to enlarge the study population in order to validate our data and the lack of an evaluation of symptoms of depression after rehabilitation treatment. Another important limitation of this pilot study is the absence of a control group (e.g., patients with the same rehabilitation but without previous stroke, patients hospitalized but without rehabilitation). Based on the presented results, we really cannot deduce if the decrease in serum serotonin is caused by PSD, hospitalization, rehabilitation, or any other cause such as nutrition or seasonal changes in blood serotonin levels.

Moreover, another limitation is that we did not measure other relevant metabolites of serotonin status as tryptophan, 5-hydroxyindoleacetic acid, kynurenine 3-hydroxykynurenine which would provide more detailed information about the actual metabolic causes behind the decrease in serotonin levels.

Nevertheless, if our results will be confirmed, the variation of serum serotonin level should be suggested as a biomarker of cognitive status in subacute stroke patients hospitalized during a rehabilitation program.

## Figures and Tables

**Table 1:** Baseline characteristics of the sample (*n* = 29).

Baseline Characteristics	Mean (±SD) or Number (%)
Age (years)	68 ± 15
Sex	
Men	13 (44.8%)
Women	16 (55.2%)
Education	10.7 (4.6)
Index stroke type	
Ischemic	23 (79.3%)
Hemorrhagic	6 (20.7%)
Affected side	
Right	11 (37.9%)
Left	18 (62.1%)
Comorbidities	
Hypertension	20 (69.0%)
Type 2 Diabetes	9 (31.0%)
Dislipidemia	5 (17.2%)
Hearth disease	12 (41.4%)
Days from index stroke to enrollment	92 ± 33
Depression	
Minimal to moderate (BDI up to 29)	18 (62.1%)
Severe (BDI equal to or higher than 30)	11 (37.9%)
Antidepressive drugs (Selective Serotonin Reuptake Inhibitors, SSRI)	16 (55.2%)
Cognitive assessment	
SDMT	21.8 ± 13.5
ROCF	20.1 ± 12.1
DS	3.6 ± 2.0
Stroop_error	4.8 ± 5.1
Stroop_time	41.0 ± 37.2
ToL_point	29.5 ± 3.5
ToL_Time	526.9 ± 263.0
ToL_errors	8.0 ± 12.4
Motor Assessment	
Modified Barthel Index (0–100)	40.7 ± 17.7
Motricity Index	30.8 ± 24.4
Fugl Meyer	16.7 ± 15.7
Serum Serotonin (ng/mL)	85.9 ± 92.4

**Table 2:** Correlation between serum serotonin levels at T0 and cognitive and motor assessment in the whole group (*n* = 29).

	Serotonin T0	
	Spearman Rho	*p*-Value
SDMT T0	−0.129	0.576
ROCF T0	0.100	0.666
DS T0	−0.086	0.712
Stroop_error T0	−0.133	0.565
Stroop_time T0	−0.189	0.413
ToL_point T0	0.583 *	**0.006**
ToL_time T0	−0.239	0.297
ToL_errors T0	−0.118	0.610
Barthel Index	0.278	0.144
Motricity Index	0.237	0.302
Fugl Meyer	0.124	0.592

* *p*-value < 0.05.

**Table 3:** Correlation of serum serotonin levels at T0 and change in serum levels of serotonin between T0 and T1 (ΔSerotonin) with change in cognitive and motor assessment score between T0 and T1 in the whole group (*n* = 29).

	Serotonin T0		ΔSerotonin	
	Spearman Rho	*p*-Value	Spearman Rho	*p*-Value
ΔSDMT	0.016	0.938	0.156	0.499
ΔROCF	−0.257	0.196	0.535 *	**0.013**
ΔDS	0.090	0.656	−0.157	0.496
ΔStroop_error	0.034	0.865	−0.165	0.476
ΔStroop_time	−0.130	0.516	0.019	0.935
ΔTol_points	−0.173	0.387	0.621 **	**0.003**
ΔTol_time	0.321	0.102	−0.619 **	**0.003**
ΔTol_error	0.378	0.052	−0.375	0.094
DeltaBI	−0.206	0.221	0.248	0.194
DeltaMI	−0.065	0.748	0.007	0.975
DeltaFM	−0.028	0.890	0.157	0.497

* *p*-value <0.05; ** *p*-value < 0.005.

**Table 4:** Correlation of change in serum levels of serotonin between T0 and T1 (ΔSerotonin) with change in cognitive and motor assessment score between T0 and T1 in patients treated and untreated with Selective Serotonin Reuptake Inhibitors (SRRI), separately.

	ΔSerotonin Treated with SSRIs (*n* = 16)		ΔSerotonin Untreated with SSRIs (*n* = 13)	
	Spearman Rho	*p*-Value	Spearman Rho	*p*-Value
ΔSDMT	0.219	0.495	0.322	0.398
ΔROCF	0.473	0.121	0.700 *	**0.036**
ΔDS	0.099	0.761	0.121	0.765
ΔStroop_error	−0.564	0.056	0.322	0.383
ΔStroop_time	−0.525	0.079	0.750 *	**0.020**
ΔTol_points	0.587 *	**0.045**	0.740 *	**0.023**
ΔTol_time	−0.503	0.095	−0.833 **	**0.005**
ΔTol_error	−0.214	0.503	−0.328	0.389
DeltaBI	0.325	0.219	−0.328	0.273
DeltaMI	0.049	0.879	0.150	0.700
DeltaFM	0.316	0.316	0.267	0.488

* *p*-value < 0.05; ** *p*-value < 0.005.

## Data Availability

The data support the findings of this study are available from the corresponding author upon reasonable request.
